# Knowledge, Attitudes, and Practices Regarding HPV Vaccination Among Healthcare Students and Professionals at a Tertiary Care Institution in Northern India

**DOI:** 10.7759/cureus.112471

**Published:** 2026-07-11

**Authors:** Himanshu Narula, Tushita Sadhu, Sulekha Nautiyal, Gaurav Chikara

**Affiliations:** 1 Microbiology, Government Medical College, Haridwar, Haridwar, IND; 2 Biochemistry, Shri Guru Ram Rai Institute of Medical and Health Sciences, Dehradun, IND; 3 Microbiology, Shri Guru Ram Rai Institute of Medical and Health Sciences, Dehradun, IND; 4 Pharmacology, All India Institute of Medical Sciences, Rishikesh, Rishikesh, IND

**Keywords:** attitude and practice, awareness of hpv, cervical cancer, healthcare workers, human papillomavirus vaccine, knowledge, medical education

## Abstract

Background: Human papillomavirus (HPV) underlies the majority of cervical cancer cases and is implicated in several other malignancies. Effective prophylactic vaccines exist, yet uptake and public understanding remain limited across India - a gap that is especially consequential among healthcare students and professionals, who are typically the first point of contact for vaccine-related counselling.

Methods: A cross-sectional survey was administered to MBBS students, nursing trainees, junior residents, and faculty at Shri Guru Ram Rai Institute of Medical and Health Sciences, Dehradun, a tertiary care institution in Northern India. This instrument was adapted from previously used HPV Knowledge, Attitudes, and Practices (KAP) questionnaires and was reviewed for content validity by experts in public health, gynaecology, and health education. Out of 240 individuals invited, 200 returned complete responses. Findings are summarised as frequencies and percentages.

Results: Every participant (200/200, 100%) had previously encountered the term "HPV," and 197/200 (98.5%) correctly linked it to cervical cancer. Notable misconceptions nonetheless emerged: 26/200 (13.0%) believed that the vaccine protects against all sexually transmitted infections, 32/200 (16.0%) thought that vaccination completely eliminates cervical cancer risk, and 58/200 (29.0%) did not realise that the vaccine is indicated for both sexes. Attitudinal responses were strongly favourable, with 188/200 (94.0%) agreeing or strongly agreeing that vaccination benefits adolescents and 159/200 (79.5%) expressing willingness to receive the vaccine.

Conclusion: Although awareness of HPV was nearly universal, important misconceptions persisted regarding HPV testing, vaccine protection, and vaccination eligibility for males. Attitudes toward HPV vaccination were generally favourable; however, HPV vaccination uptake was low. Strengthening HPV-focused education and addressing barriers to vaccine access may support prevention efforts among healthcare students and professionals.

## Introduction

More than 200 human papillomavirus (HPV) types have been identified. HPV-16 and HPV-18 account for approximately 70% of cervical cancer cases and also contribute to ano-genital and oropharyngeal cancers [1]. Cervical cancer ranks fourth among cancers affecting women globally, with the highest burden on low- and middle-income countries (LMICs), where constrained screening capacity often delays diagnosis and worsens outcomes [2].

Several prophylactic HPV vaccine formulations bivalent, quadrivalent, and nonavalent have shown strong protective effects against HPV acquisition and the development of precancerous cervical changes. Major health bodies, including the World Health Organization and the United States Centers for Disease Control and Prevention, advocate for including HPV vaccination into routine national immunization schedules. School-based vaccination efforts in countries such as Australia and the United Kingdom have driven down HPV prevalence substantially. LMICs, by contrast, continue to grapple with entrenched barriers - circulating misinformation, cultural reluctance, prohibitive vaccine costs, supply constraints, and gaps in public knowledge [2,3].

India alone contributes close to a quarter of the world's cervical cancer deaths, with annual figures exceeding 120,000 new diagnoses and 60,000 fatalities. HPV vaccination has not yet achieved widespread adoption nationally. This is particularly evident in Northern India, where uneven healthcare infrastructure and variable health literacy between urban and rural populations have produced inconsistent awareness of HPV and HPV vaccination [3]. In this context, examining what healthcare students and professionals know, believe, and practice regarding HPV is especially relevant, given their role as front-line educators and counsellors for the wider public [4]. Healthcare students and professionals occupy an important role in shaping public understanding of HPV-related disease and prevention. However, evidence suggests that misconceptions and inconsistent vaccination-promoting behavior can persist even within this relatively well-educated group [5].

Medical and nursing students are future healthcare providers; therefore, their grasp of HPV prevention has long-term implications for population-level uptake. Mapping their existing knowledge, attitudes, and practices offers a window into how well current training pipelines are equipping future providers to address a largely preventable disease. Despite increasing global attention to HPV vaccination, dedicated research examining the KAP profile of healthcare students and professionals specifically within Northern India remains sparse [3].

Study objectives

The primary objective of this study was to assess the knowledge, attitudes, and practices regarding HPV infection and HPV vaccination among healthcare students and professionals at a tertiary care institution in Northern India.

The specific objectives were to assess participants’ knowledge of HPV infection, HPV testing, and HPV vaccination; evaluate attitudes toward HPV vaccination, school-based HPV education, and cervical cancer screening; describe HPV vaccination uptake and preventive practices among participants; and identify perceived barriers to HPV vaccination and recommendations.

## Materials and methods

Study design and setting

The study followed a cross-sectional, questionnaire-based quantitative design intended to capture a snapshot of HPV-related knowledge, attitudes, and practice among healthcare students and professionals. Data collection took place at Shri Guru Ram Rai Institute of Medical and Health Sciences, Dehradun, a tertiary care center in Northern India serving both urban and semi-urban populations. Four respondent groups were targeted: MBBS students, nursing students, junior residents, and faculty members spanning the full educational and professional hierarchy within the institution and thereby capturing variation in HPV-related understanding across training stages.

Sampling and eligibility

A non-probability convenience sampling method was used because the study was conducted at a single tertiary care institution and aimed to obtain an exploratory assessment of HPV-related knowledge, attitudes, and practices among different healthcare groups. During the two-month data-collection period, eligible MBBS students, nursing trainees, junior residents, and faculty members were approached through classroom sessions, departmental visits, and institutional communication channels. Participation was voluntary, and no incentives were offered.

Participants were included if they were currently enrolled or employed at the institution, were willing to participate, and could read and complete the English-language questionnaire. Individuals who submitted incomplete or duplicate questionnaires were excluded. Of the 240 individuals approached, 200 submitted complete questionnaires and were included in the final analysis. As this was an exploratory institution-based survey using convenience sampling, no formal a priori sample-size calculation was performed. Instead, all eligible individuals available and willing to participate during the study period were invited to complete the questionnaire.

Study instrument

Data were gathered through a structured, self-administered questionnaire organised into three sections consistent with the standard KAP survey format. The first section used closed* Yes/No/Do Not Know* items to probe knowledge of HPV transmission routes, its link to cervical and other cancers, testing procedures, and vaccine availability and eligibility. The second section presented five attitude statements rated on a five-point Likert scale from "strongly agree" to "strongly disagree," capturing views on vaccine necessity, safety, and applicability across genders. The third section assessed HPV-related preventive and clinical practices, including vaccination status, cervical cancer screening practices, HPV vaccination counselling, and perceived barriers to vaccine uptake and recommendation. This instrument was adapted from previously used HPV KAP questionnaires and was reviewed for content validity by experts in public health, gynecology, and health education. The complete questionnaire is provided in Appendices 1-3.

Data collection

Fieldwork spanned two months and combined both online and paper-based survey formats. Where feasible, paper questionnaires were distributed directly within classroom and departmental settings. No financial or material incentives were provided, and participation remained entirely voluntary throughout. Respondents completed the survey independently to preserve the integrity and authenticity of their individual answers.

Statistical analysis

Survey responses were entered into Microsoft Excel (Microsoft Corp., USA) and analysed using SPSS version 26.0 (IBM Corp., Armonk, NY, USA). Participant characteristics and item-level responses related to knowledge, attitudes, and practices were summarised using frequencies and percentages. Practice-related items were analysed descriptively because some questions were applicable only to specific participant subgroups, including female respondents and participants with patient-contact responsibilities. Therefore, a single composite Practice Score applicable to all participants could not be constructed, and formal analyses of associations between knowledge, attitudes, and practices were not performed.

Ethical approval

Approval for the study protocol was granted by the Institutional Ethics Committee of the host institution (reference no. SGRR/IEC/01/23). Throughout data collection, the research team upheld core ethical commitments: participation was voluntary, informed consent was documented, and respondent confidentiality and anonymity were preserved. Every participant signed a written consent form before any data were gathered.

## Results

Participant characteristics

A total of 200 respondents completed the survey (out of 240 approached). The mean age was 29.3 ± 11.8 years; more than half of the participants (106, 53.0%) were in the 18-24-year age group, 38 (19.0%) in the 25-34 range, and 56 (28.0%) aged 35 years or above. The cohort was predominantly female (111, 55.5%), with 89 male respondents (44.5%). Faculty formed the largest single professional category (58, 29.0%), followed by nursing trainees (51, 25.5%), MBBS students (50, 25.0%), and junior residents (41, 20.5%). Demographic details are presented in Table 1.

**Table 1:** Participant characteristics (n = 200) SD: standard deviation, MBBS: Bachelor of Medicine and Bachelor of Surgery

Variable	Frequency (n)	Percentage (%)
Age (in years)		
Mean ± SD	29.3 ± 11.8	—
18–24 years	106	53.0
25–34 years	38	19.0
35 years and above	56	28.0
Sex		
Female	111	55.5
Male	89	44.5
Professional role		
Faculty	58	29.0
Nursing trainee	51	25.5
MBBS student	50	25.0
Junior resident	41	20.5

Knowledge of HPV infection

Every one of the 200/200 (100%) respondents reported prior awareness of HPV. Nearly all respondents (197/200, 98.5%) recognized HPV as a cause of cervical cancer, with only three uncertain. The asymptomatic, long-persisting nature of HPV infection was correctly identified by 194/200 (97.0%) participants. Responses concerning risk factors and transmission routes were somewhat more variable. A total of 191/200 respondents (95.5%) correctly recognized multiple sexual partners as a risk factor for HPV infection; six (3.0%) were unsure, and three (1.5%) disagreed. On the question of symptom visibility, 182/200 (91.0%) participants correctly understood that HPV infection need not present visible signs, although 12 (6.0%) held the opposite, incorrect belief. Knowledge regarding male susceptibility proved weaker: 171/200 (85.5%) respondents correctly rejected the notion that men cannot contract HPV, yet 23 (11.5%) endorsed this misconception. Awareness that HPV causes genital warts was high (191/200, 95.5%). However, 17 participants (8.5%) believed that antibiotics could cure HPV, and 14 (7.0%) were unsure. These findings appear in Table 2.

**Table 2:** Responses to HPV infection knowledge items (n = 200) HPV: human papilloma virus. Values represent the number of respondents.

Knowledge question	Do not know	No	Yes
Before today, had you heard of human papillomavirus (HPV)?	0	0	200
HPV can cause cervical cancer.	3	0	197
A person could have an HPV infection for many years without knowing it.	3	3	194
Having many sexual partners increases the risk of HPV infection.	6	3	191
HPV infection always has visible signs or symptoms.	6	182	12
Men cannot get infected with HPV.	6	171	23
HPV can cause genital warts.	6	3	191
HPV can be cured with antibiotics.	14	169	17

Knowledge of HPV testing

Familiarity with HPV testing was reported by 194/200 (97.0%) participants. When asked whether a positive HPV test guarantees future cervical cancer, 172 respondents (86.0%) disagreed, 14 (7.0%) agreed, and 14 (7.0%) were unsure. For the item asking whether HPV testing is used to indicate whether HPV vaccination is needed, 46/200 (23.0%) responded correctly, 125/200 (62.5%) responded incorrectly, and 29/200 (14.5%) were unsure. For the item concerning a negative HPV test and cervical cancer risk, 137/200 respondents (68.5%) responded correctly, 43 (21.5%) responded incorrectly, and 20 (10.0%) were unsure. Responses are presented in Table 3.

**Table 3:** Responses to HPV testing knowledge items (n = 200) HPV: human papillomavirus. Values represent the number of respondents.

Knowledge question	Do not know	No	Yes
Have you heard of testing done for HPV infection?	3	3	194
If a woman tests positive for HPV, she will definitely get cervical cancer.	14	172	14
HPV testing is used to indicate whether the HPV vaccine is needed.	29	125	46
If an HPV test shows a woman does not have HPV, her risk of cervical cancer is low.	20	43	137

Knowledge of HPV vaccination

**Figure 1:**
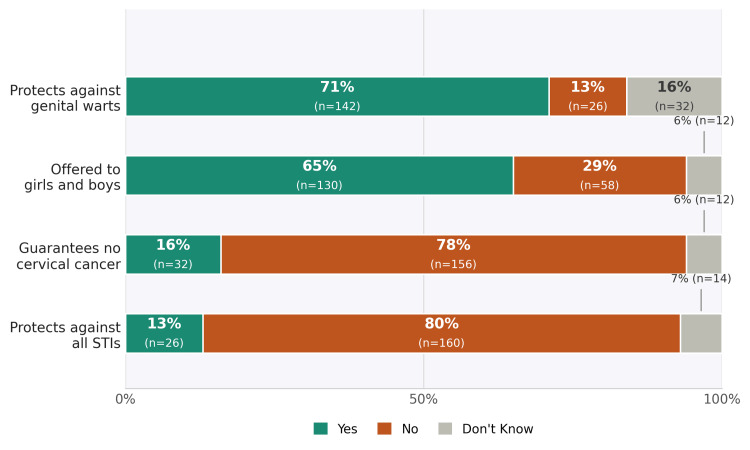
Responses to HPV vaccination knowledge items (n = 200) Source: Generated in Microsoft Excel

Understanding of what the HPV vaccine does and does not protect against varied across items. Most respondents, 160/200 (80.0%), correctly recognised that the vaccine does not protect against all sexually transmitted infections, although 26 (13.0%) mistakenly believed it does and 14 (7.0%) were unsure. A related misconception concerned absolute protection from cervical cancer: 156/200 respondents (78.0%) correctly understood that vaccination does not eliminate this risk entirely, while 32 (16.0%) incorrectly assumed complete protection. Awareness that the vaccine is indicated for both sexes was moderate - 130 respondents (65.0%) answered correctly, whereas 58 (29.0%) believed it was intended for girls only, and 12 (6.0%) were unsure. Knowledge that the vaccine also protects against genital warts was held by 142 participants (71.0%), with 32 (16.0%) uncertain and 26 (13.0%) incorrectly denying this protective effect. Complete details are shown in Figure 1.

Attitudes towards HPV vaccination

Attitudinal responses were consistently favourable across every item assessed. On the question of whether vaccination benefits adolescents of either gender, 147/200 respondents (73.5%) strongly agreed and a further 41 (20.5%) agreed, with no outright disagreement recorded and only 12 (6.0%) remaining neutral. Support for introducing HPV education at the school level was similarly strong, with 156/200 (78.0%) strongly agreeing, 41 (20.5%) agreeing, and just three (1.5%) neutral responses. When asked whether HPV vaccination is unnecessary because a Pap smear can be performed to rule out cervical cancer, 177/200 respondents (88.5%) disagreed or strongly disagreed. Six respondents (3.0%) agreed, three (1.5%) strongly agreed, and 14 (7.0%) were neutral.

Endorsement of Pap smear screening as a recommendation was likewise strong, with 125/200 respondents (62.5%) strongly agreeing and 58 (29.0%) agreeing. Personal intention to receive HPV vaccination was reported by 98 respondents (49.0%) who strongly agreed, and 61 (30.5%) who agreed; 32 (16.0%) were neutral, three (1.5%) disagreed, and six (3.0%) strongly disagreed. The complete attitudinal dataset is presented in Table 4.

**Table 4:** Attitudes toward HPV vaccination among the respondents (n = 200) HPV: human papillomavirus. Values represent the number of respondents. Five-point Likert scale: strongly agree to strongly disagree.

Attitude statement	Agree	Disagree	Neutral	Strongly agree	Strongly disagree
Getting the HPV vaccine will be beneficial to a teenage girl or boy for their future health.	41	0	12	147	0
Education on HPV should be given at the school level.	41	0	3	156	0
HPV vaccination is NOT necessary because a Pap smear can be done to rule out cervical cancer.	6	96	14	3	81
I want to recommend having a proper Pap smear performed for cervical cancer screening.	58	0	14	125	3
I want to get vaccinated for HPV.	61	3	32	98	6

Personal preventive practice

**Figure 2:**
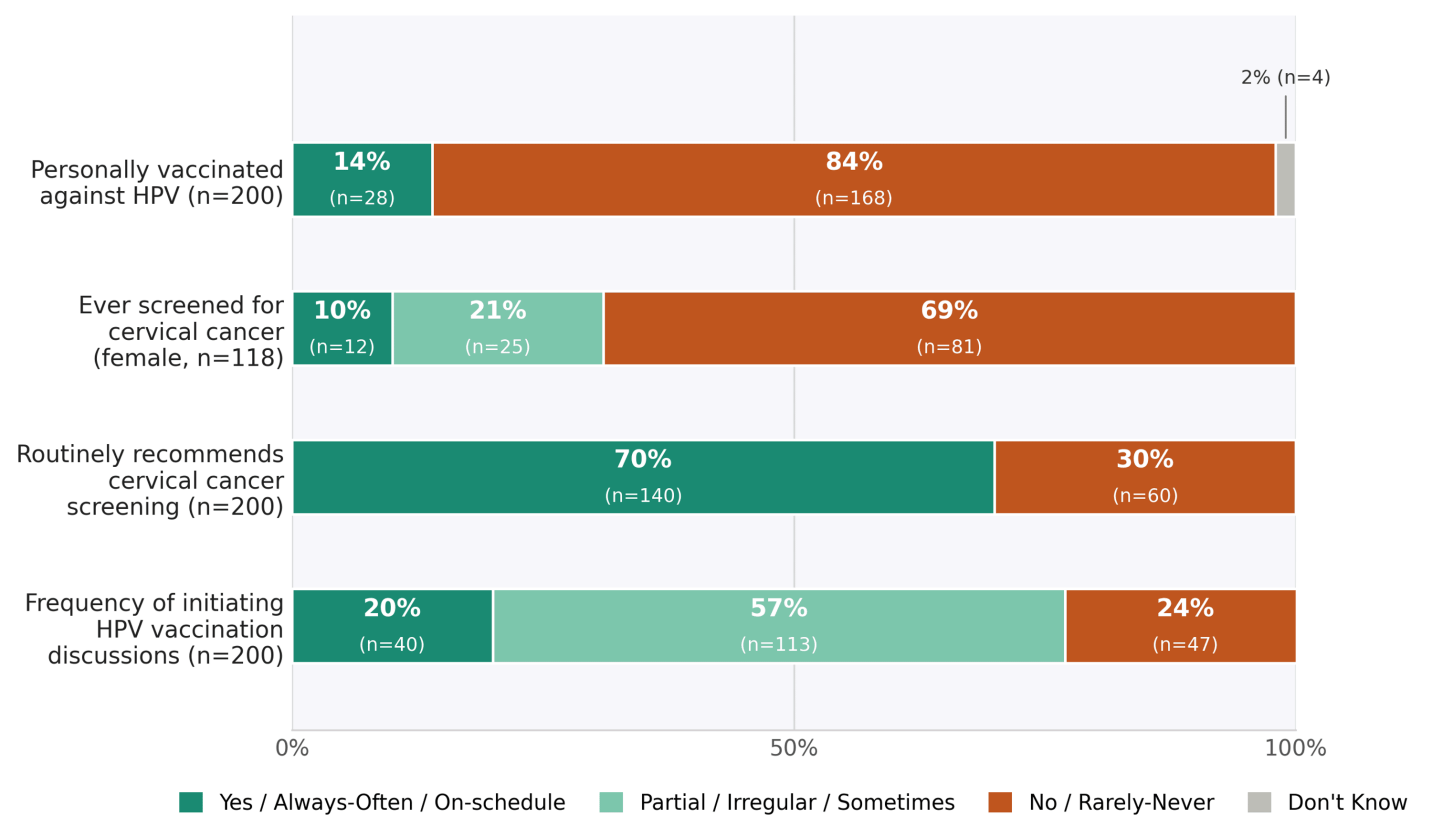
HPV-related preventive practices among the respondents Source: Created using Microsoft Excel

Personal HPV vaccination uptake was low: 28/200 (14.0%) respondents had received at least one dose, including 14/200 (7.0%) who had completed vaccination and 14/200 (7.0%) who had received a partial schedule. Moreover, 168/200 (84.0%) remained unvaccinated and 4/200 (2.0%) were unsure of their vaccination status. The remaining practice-related responses are summarized in Figure 2.

Clinical practice: patient care and advocacy

Only 39/200 (19.5%) respondents reported consistently initiating HPV vaccination discussions with eligible patients (defined as answering "always" or "often"), whereas 140/200 (70.0%) routinely recommended cervical cancer screening to eligible patients, indicating a clear asymmetry between these two forms of preventive advocacy. Among respondents who reported rarely or never discussing HPV vaccination (47/200, 23.5%), the most frequently cited barriers were that HPV vaccination counselling fell outside their scope of practice (16/47, 34.0%) and that patients could not afford the vaccine (14/47, 29.8%). Less commonly reported barriers included lack of time during consultations (6/47, 12.8%), insufficient information to provide counselling (6/47, 12.8%), and concerns regarding cultural or parental resistance (5/47, 10.6%).

Regarding personal preventive practices, 27/200 (13.5%) respondents reported receiving at least one dose of HPV vaccine, including 13/200 (6.5%) who had completed all recommended doses and 14/200 (7.0%) who had received partial vaccination. Most respondents remained unvaccinated (169/200, 84.5%), while 4/200 (2.0%) were uncertain of their vaccination status. Among unvaccinated respondents (n = 169), the most commonly reported barriers were high cost (58/169, 34.3%), not perceiving themselves to be at risk (37/169, 21.9%), and waiting for more information (34/169, 20.1%). Among female respondents (n = 118), only 12/118 (10.2%) reported cervical cancer screening within the recommended timeframe, 25/118 (21.2%) had been screened but not regularly, and 81/118 (68.6%) had never undergone cervical cancer screening. Detailed item-level responses are presented in Table 5.

**Table 5:** Practice questionnaire item-level responses (n = 200) HPV: human papillomavirus. Values represent the number of respondents and percentage of the relevant denominator.

Practice item / response category	n	%
Personal preventive practice		
Have you ever received the HPV vaccine? (P1)		
Yes, completed all doses	13	6.5
Yes, partially vaccinated	14	7.0
No	169	84.5
Do not know / cannot remember	4	2.0
Most common barrier to vaccination, among unvaccinated (P2, n = 169)		
High cost	58	34.3
Do not consider self at risk	37	21.9
Waiting for more information	34	20.1
Lack of availability/access	21	12.4
Fear of side effects/safety concerns	19	11.2
Ever screened for cervical cancer (P3, female respondents only, n = 118)		
Yes, within the recommended timeframe	12	10.2
Yes, but not regularly	25	21.2
No, never	81	68.6
Clinical practice (patient care and advocacy)		
Frequency of initiating HPV vaccination discussions with patients (P4, n = 200)		
Always	10	5.0
Often	29	14.5
Sometimes	114	57.0
Rarely	38	19.0
Never	9	4.5
Routinely recommends cervical cancer screening to eligible patients (P5, n = 200)		
Yes	140	70.0
No	60	30.0
Main reason for not discussing HPV vaccination, among those answering rarely/never to P4 (P6, n = 47)		
Outside my scope of practice / not my specialty	16	34.0
Patients cannot afford it.	14	29.8
Lack of time during consultations	6	12.8
Insufficient information to counsel properly	6	12.8
Fear of cultural/parental pushback	5	10.6

## Discussion

This study evaluated HPV-related knowledge, attitudes, and practices among healthcare students and professionals at a tertiary care institution in Northern India. Although awareness of HPV and its association with cervical cancer was high, important misconceptions persisted regarding vaccine effectiveness, HPV testing, and gender-inclusive vaccination. Similar patterns have been documented among healthcare students and professionals across diverse settings, where high awareness frequently coexists with gaps in vaccine-specific knowledge and preventive practices [5-7].

Knowledge deficits were particularly evident in the interpretation of HPV testing, with less than one-quarter of respondents correctly understanding its role in vaccination decisions. Previous studies have identified similar deficiencies among both students and practicing physicians, highlighting persistent uncertainty regarding screening pathways, vaccine indications, and interpretation of test results [8,9]. Misconceptions regarding complete protection following vaccination, treatment of HPV infection with antibiotics, and male eligibility for vaccination further suggest that awareness alone is insufficient to ensure comprehensive understanding of HPV prevention.

Attitudes toward HPV vaccination were overwhelmingly positive, with most respondents supporting vaccination, school-based HPV education, and cervical cancer screening. Educational interventions have consistently demonstrated improvements in HPV-related knowledge and attitudes among healthcare students, supporting the integration of structured HPV education within healthcare curricula [10]. However, favorable attitudes did not consistently translate into practice. Only 14% of respondents had received at least one dose of HPV vaccination, with cost emerging as the most commonly reported barrier. Similar studies have identified affordability and accessibility as major determinants of vaccine uptake in low- and middle-income settings [11,12].

Practice-related responses were interpreted descriptively because some questionnaire items were applicable only to particular participant subgroups, including female respondents and those with patient-contact responsibilities. Therefore, a direct comparison of a composite Practice Score across gender and professional categories was not considered appropriate. Nevertheless, the reported responses suggest a gap between favorable attitudes and HPV-related preventive practices, including personal HPV vaccination uptake and vaccination-related counselling, a pattern also reported in other healthcare and non-healthcare populations [13-14].

The discrepancy between high HPV-related knowledge and limited preventive practices may be interpreted using behavioral science frameworks such as the Health Belief Model and the Theory of Planned Behavior [15,16]. Knowledge alone may not result in HPV vaccination or vaccine counselling unless individuals also perceive themselves or their patients to be at risk, believe that HPV vaccination offers meaningful benefits, and perceive barriers, such as cost, access, social norms, and scope-of-practice concerns, as manageable [15-17]. Similarly, favorable attitudes may not translate into behavior when perceived behavioral control is limited by financial constraints, lack of institutional vaccine availability, inadequate opportunities for counselling, or uncertainty regarding professional responsibility [16,17]. These frameworks may help explain why awareness and positive attitudes did not consistently correspond with HPV vaccination uptake or preventive practices in the present study. However, these factors were not directly measured and should be explored in future longitudinal and qualitative research.

The persistence of positive attitudes alongside suboptimal vaccine uptake mirrors findings reported across healthcare and non-healthcare populations worldwide [13-14]. Collectively, these findings indicate that barriers to HPV vaccination extend beyond knowledge alone and include financial, structural, and practice-related factors. Integrating HPV vaccination modules into undergraduate and postgraduate curricula, promoting institution-based awareness programs, and improving vaccine accessibility may be associated with improved knowledge and preventive practices, although this requires evaluation in intervention studies.

India has recently initiated nationwide HPV vaccination efforts, with the Government of India launching an HPV vaccination campaign for 14-year-old girls [18]. These initiatives may improve access over time; however, their impact will depend on sustained vaccine availability, affordability, community awareness, and healthcare-provider preparedness. Strengthening HPV-related education among healthcare students and professionals may support effective implementation of these prevention efforts.

Limitations

Several limitations should be considered when interpreting these findings. Recruitment relied on convenience sampling at a single tertiary care institution, which limits generalizability beyond the study population. Although 200 of 240 approached individuals provided complete responses, the cross-sectional design does not permit causal inferences regarding knowledge, attitudes, HPV vaccination uptake, or preventive practices. The use of a self-administered questionnaire may have introduced self-reporting bias because participants’ vaccination status, attitudes, and practices could not be independently verified. Social desirability bias may also have led respondents to report more favorable attitudes toward HPV vaccination or more appropriate preventive practices than they actually held or performed. Accordingly, the findings may not be generalizable to healthcare students and professionals in other regions, institutions, or community-based settings.

In addition, some practice-related items were applicable only to specific subgroups, such as female respondents and participants with patient-contact responsibilities. Therefore, practice items were analyzed descriptively rather than through direct comparison of a composite Practice Score across gender or professional categories. The questionnaire was not designed to generate uniformly applicable composite knowledge, attitude, and practice measures; consequently, formal analyses of relationships among these domains could not be undertaken. Although the questionnaire underwent expert content review, it was not formally pilot tested, and internal-consistency reliability statistics such as Cronbach’s alpha were not calculated; therefore, its measurement properties could not be fully established. Future multicenter studies using probability sampling, larger samples, validated uniformly applicable instruments, and longitudinal or qualitative designs would help clarify factors associated with HPV vaccination uptake and advocacy.

Clinical implications

The findings highlight misconceptions regarding HPV treatment, vaccine effectiveness, and male eligibility. Integrating HPV-focused educational content into healthcare curricula and improving vaccine accessibility may help improve vaccine uptake and advocacy.

## Conclusions

This cross-sectional study found that healthcare students and professionals at a tertiary care institution in Northern India had high general awareness of HPV and its association with cervical cancer. However, important misconceptions persisted regarding vaccine scope, interpretation of HPV testing, and eligibility of males for vaccination. Although attitudes toward HPV vaccination were generally favourable, HPV vaccination uptake remained low across the surveyed groups.

Given the cross-sectional design, convenience sampling, and single-institution setting, these findings should be interpreted cautiously and may not be generalisable to all healthcare students and professionals in Northern India. The observed gap between favourable attitudes and limited HPV-related preventive practices may reflect barriers beyond knowledge alone, including cost, accessibility, institutional availability, and perceived professional responsibilities. Larger multicentre studies using validated instruments and longitudinal or qualitative designs are needed to examine factors associated with HPV vaccination uptake and advocacy.

## Appendices

Appendix 1

**Table 6:** Knowledge of HPV infection

#	Statement	Yes	No	Do not know
1	Before today, had you heard of human papillomavirus (HPV)?	□	□	□
2	HPV can cause cervical cancer.	□	□	□
3	A person could have an HPV infection for many years without knowing it.	□	□	□
4	Having many sexual partners increases the risk of HPV infection.	□	□	□
5	HPV infection always has visible signs or symptoms.	□	□	□
6	Men cannot get infected with HPV.	□	□	□
7	HPV can cause genital warts.	□	□	□
8	HPV can be cured with antibiotics.	□	□	□

**Table 7:** Knowledge of HPV testing

#	Statement	Yes	No	Do not know
1	Have you heard of testing done for HPV infection?	□	□	□
2	If a woman tests positive for HPV, she will definitely get cervical cancer.	□	□	□
3	HPV testing is used to indicate whether the HPV vaccine is needed.	□	□	□
4	If an HPV test shows a woman does not have HPV, her risk of cervical cancer is low.	□	□	□

**Table 8:** Knowledge of HPV vaccination

#	Statement	Yes	No	Do not know
1	HPV vaccination protects against all sexually transmitted infections.	□	□	□
2	HPV vaccination completely eliminates the risk of cervical cancer.	□	□	□
3	HPV vaccination is recommended for both males and females.	□	□	□
4	HPV vaccination can help prevent genital warts.	□	□	□

Appendix 2

**Table 9:** Attitude questionnaire

#	Statement	Strongly agree	Agree	Neutral	Disagree	Strongly disagree
1	Getting the HPV vaccine will be beneficial to a teenage girl or boy for their future health.	□	□	□	□	□
2	Education on HPV should be given at the school level.	□	□	□	□	□
3	HPV vaccination is NOT necessary because a Pap smear can be done to rule out cervical cancer.	□	□	□	□	□
4	I want to recommend having a proper Pap smear performed for cervical cancer screening.	□	□	□	□	□
5	I want to get vaccinated for HPV.	□	□	□	□	□

Appendix 3: Practice Questionnaire

**Table 10:** Personal HPV vaccination status and barriers to vaccination HPV: human papillomavirus

No.	Question	Response option	□
P1	Have you received the HPV vaccine?	Yes, completed vaccination (all recommended doses)	□
		Yes, partially vaccinated (at least one dose, but not the full schedule)	□
		No	□
		Not sure	□
P2	If you have not been vaccinated, what is the main reason? Answer only if applicable.	High cost	□
		Do not consider myself at risk	□
		Waiting for more information	□
		Concern about side effects	□
		Vaccine not available in my area	□
		Other: _________________________________	□

**Table 11:** Personal cervical cancer screening history

No.	Question	Response option	□
P3	Have you ever undergone cervical cancer screening? For female respondents only — please skip if not applicable.	Regularly, as per recommended guidelines	□
		Occasionally / irregularly	□
		Never	□

**Table 12:** Frequency of HPV vaccination discussions and barriers to clinical counselling HPV: human papillomavirus

No.	Question	Response option	□
P4	How often do you initiate discussions about HPV vaccination with eligible patients or parents?	Always	□
		Often	□
		Sometimes	□
		Rarely	□
		Never	□
P5	If you rarely or never discuss HPV vaccination with patients, what is the main reason? Answer only if applicable.	Lack of time during consultations	□
		Insufficient knowledge about HPV vaccination	□
		Scope-of-practice concerns	□
		Lack of confidence in counselling	□
		Patient or parent reluctance	□
		Other: _________________________________	□

**Table 13:** Routine recommendation of cervical cancer screening

No.	Question	Response option	□
P6	Do you routinely recommend cervical cancer screening to eligible female patients?	Yes	□
		No	□
